# Comparison of Corn Stover Pretreatments with Lewis Acid Catalyzed Choline Chloride, Glycerol and Choline Chloride-Glycerol Deep Eutectic Solvent

**DOI:** 10.3390/polym13071170

**Published:** 2021-04-06

**Authors:** Yuan Zhu, Benkun Qi, Xinquan Liang, Jianquan Luo, Yinhua Wan

**Affiliations:** 1 School of Light Industry and Food Engineering, Guangxi University, Nanning 530004, China; 1816301040@st.gxu.edu.cn; 2 State Key Laboratory of Biochemical Engineering, Institute of Process Engineering, Chinese Academy of Sciences, Beijing 100190, China; jqluo@ipe.ac.cn (J.L.); yhwan@ipe.ac.cn (Y.W.); 3 School of Chemical Engineering, University of Chinese Academy of Sciences, Beijing 100049, China

**Keywords:** lewis acid, choline chloride, glycerol, deep eutectic solvent, lignocellulosic pretreatment

## Abstract

Herein, corn stover (CS) was pretreated by less corrosive lewis acid FeCl_3_ acidified solutions of neat and aqueous deep eutectic solvent (DES), aqueous ChCl and glycerol at 120 °C for 4 h with single FeCl_3_ pretreatment as control. It was unexpected that acidified solutions of both ChCl and glycerol were found to be more efficient at removing lignin and xylan, leading to higher enzymatic digestibility of pretreated CS than acidified DES. Comparatively, acidified ChCl solution exhibited better pretreatment performance than acidified glycerol solution. In addition, 20 wt% water in DES dramatically reduced the capability of DES for delignification and xylan removal and subsequent enzymatic cellulose saccharification of pretreated CS. Correlation analysis showed that enzymatic saccharification of pretreated CS was highly correlated to delignification and cellulose crystallinity, but lowly correlated to xylan removal. Recyclability experiments of different acidified pretreatment solutions showed progressive decrease in the pretreatment performance with increasing recycling runs. After four cycles, the smallest decrease in enzymatic cellulose conversion (22.07%) was observed from acidified neat DES pretreatment, while the largest decrease (43.80%) was from acidified ChCl pretreatment. Those findings would provide useful information for biomass processing with ChCl, glycerol and ChCl-glycerol DES.

## 1. Introduction

Lignocellulosic biomass, endowed with characteristics like wide availability, renewability and cheap cost, has been considered as an eco-friendly alternative to petrochemical sources. In the past decades, research efforts have been devoting to conversion of these lignocelluloses to value-added products including fine chemicals, functional materials and biofuels, with the aim to compete with petroleum-derived equivalents in a sustainable and eco-efficient production process [1]. Lignocellulosic feedstock is mainly composed of cellulose, hemicellulose and lignin, of which cellulose and hemicellulose are two polysaccharide fractions, accounting for more than 50% of lignocellulosic composition [2]. High-efficient extraction and utilization of carbohydrate plays decisive roles in lignocellulosic bioconversion, which consists of three independent steps, i.e., pretreatment, enzymatic hydrolysis and microbial fermentation [3,4]. Pretreatment to deconstruct the recalcitrant structure of cellulosic feedstock is believed to be a prerequisite step for enzymatic depolymerization of polysaccharides in lignocellulose with commercially available (hemi)cellulolytic enzymes. In light of the importance of pretreatment, researchers around the world have developed a wide variety of pretreatment methods and those include physical (milling, microwave, etc.), chemical (acid, alkali, ionic liquid, etc.), physicochemical (steam explosion, ammonia explosion, etc.) and biological (fungi, laccase, etc.) [5,6]. Albeit with intensive research, there are still some tough challenges remain to be addressed for some of aforementioned conventional pretreatments, such as long processing time (up to days), high-energy consumption, poor pretreatment performance, corrosion to the equipment, high cost of waste pretreatment or chemical recovery, etc. [7] 

In the current context of green chemistry, pretreatment of lignocellulosic biomass with renewable and sustainable processes under mild conditions is increasingly receiving attention of scientific and industrial community [8]. Among the recently newly developed pretreatment methods, green solvent deep eutectic solvent (DES) emerges and stands out due to its unique properties like easy preparation without purification and waster generation under mild conditions, cheap cost, low/no toxicity and high biodegradability [9]. DES is composed of two or three components that can self-associate to form homogeneous solvents through hydrogen bond interactions between hydrogen bond donors (HBD) and hydrogen bond acceptors (HBA), forming a eutectic mixture with melting point lower than each of its components [10]. DES exhibits physicochemical properties similar to ionic liquid but are generally cheaper and easier to prepare and are more environmentally benign [11]. DES are widely being exploited for pretreatment of cellulosic materials due to its excellent capability of solubilizing lignin and increasing the accessibility of cellulose [12,13,14,15]. Procentese, et al. [16] compared the energy consumption of DES pretreatment with that of the most commonly used pretreatment processes, they concluded that DES pretreatment required about 28% and 72% less energy than NaOH pretreatment and steam explosion, respectively, demonstrating the great potential and feasibility of DES in processing lignocelluloses. 

The mass-produced and sustainable choline chloride (ChCl) is the most found HBA for DES synthesis. The glycerol, a type of commonly used polyalcohol based HBD, is generated in large quantity as a by-product of biodiesel industry. Expectedly, application of DES, prepared from low-cost ChCl and glycerol, for biomass pretreatment could reduce pretreatment cost to some extent in comparison to other DES solvents. However, available literature showed that single DES prepared from ChCl and glycerol was ineffective in biomass pretreatment for removing lignin [17,18,19]. Therefore, tuning the properties of ChCl-glycerol DES for improved pretreatment performance remains to be resolved. Chen, et al. [20] acidified the aqueous ChCl-glycerol DES with H_2_SO_4_ and used the acidified solution for switchgrass pretreatment at 120 °C for 1 h. They found that the removals of xylan and lignin increased, respectively, from 6.91% and 17.72% for single DES pretreatment to 78.77% and 56.62% for acidified DES pretreatment, clearly demonstrating the strongly positive effect of acid on improving the DES pretreatment performance. In order to reduce the corrosion issues and environmental footprint caused by strong mineral acids, Wang, et al. [21] replaced H_2_SO_4_ with five lewis acids (ZnCl_2_, FeCl_2_, AlCl_3_, CuCl_2_ and FeCl_3_) for acidification of ChCl-glycerol DES. They found FeCl_3_ acidified DES pretreatment of hybrid *pennisetum* (120 °C, 6 h) showed the best performance, removing 93.63% hemicellulose and 78.88% lignin, among the tested lewis acids. By conducting literature search, it was found that biomass pretreatment with combined acidic catalyst and individual component of ChCl-glycerol DES, i.e., ChCl or glycerol, can also remarkably improve the pretreatment performance in terms of delignification and hemicellulose removal compared to their counterparts without acid addition. Corrosive strong acid assisted glycerol pretreatments have been widely used for pretreating a variety of cellulosic materials [22]. However, lewis acid catalyzed glycerol pretreatment was seldom reported. Tang, et al. [23] pretreated rice straw with 90 wt% aqueous glycerol combined with 0.08 mol/L AlCl_3_ at 146.8 °C for 20 min. They found that removal of lignin and hemicellulose reached 83% and 94%, respectively, and enzymatic hydrolysis of pretreated biomass yielded to 74% glucose yield. Santana, et al. [24] reported 61.3% delignification and 93.9% hemicellulose removal after processing water hyacinth with pure glycerol in the presence of 0.1 mol/L FeCl_3_ at 220 °C for 10 min. As far as acid-facilitated ChCl pretreatment was concerned, Chen, et al. [25] reported 76.17% xylan removal and 51.10% delignification after switchgrass pretreatment with 75% ChCl in combination with 1.0% H_2_SO_4_ at 120 ^o^C for 25 min. To our best knowledge, there is no report on lignocellulose pretreatment with lewis acid-catalyzed ChCl solution. 

In this work, corn stover (CS) was pretreated with lewis acid FeCl_3_-acidified solutions of neat and aqueous ChCl-glycerol DES, glycerol and ChCl with single FeCl_3_ as pretreatment control for further cellulose saccharification, aiming to understand the roles that acidified individual components of DES played in acidified DES pretreatment. After pretreatment, the pretreatment slurries were separated into solid stream rich in cellulose and acidified liquid stream containing pretreatment agents, carbohydrate and pretreatment by-products. The cellulose-rich solid fractions were analyzed for chemical compositions and characterized by Fourier transform infrared analyzer (FT-IR), scanning electron microscopy (SEM) and X-ray diffraction (XRD). The liquid fractions were quantified for contents of carbohydrate and by-products using high-performance liquid chromatography (HPLC). The reusability of different acidified pretreatment solutions was evaluated and compared in detail in order to save pretreatment cost. All in all, a better understanding of contribution that individual component of DES made in DES pretreatment would provide useful insights into action mechanism of DES for lignocellulosic pretreatment.

## 2. Materials and Methods

### 2.1. Materials

Biomass CS were collected from a farm in Hengshui, China. CS were washed with tap water to remove impurities and then dried at 60 °C to constant weight. The dried CS were milled to powders with particle sizes varying between 60–80 mesh. The chemical compositions of CS based on dry weight were as follows: 32.39% glucan, 22.94% xylan, 21.91% lignin (20.33% acid-insoluble lignin and 1.58% acid-soluble lignin) and 4.61% ash. Cellulase with a filter paper activity (FPA) of 112 FPU/mL and β-glucosidase with a cellobiase unit (CBU) of 118 CBU/mL were purchased from Sunson Industrial Group Co., Ltd., Beijing, China. All the chemicals used in the present study were of analytical grade and purchased from China Sinopharm Chemical Reagent Co., Ltd. (Beijing, China) if not specified otherwise.

### 2.2. Preparation of Pretreatment Solutions

FeCl_3_ mediated ChCl-glycerol DES was prepared as follows: the ternary mixture consisting of ChCl, glycerol and FeCl_3_·6H_2_O at a mole ratio of 62:124:1 was stirred in an oil bath at 80 °C until a transparent liquid was obtained. Then, the mixture was dried under vacuum 80 °C for 24 h to remove water. The mass ratio of FeCl_3_ in ChCl-glycerol DES was calculated to be 1.32 wt%. Aqueous solution of as-prepared FeCl_3_-acidified DES contained 20 wt% deionized (DI) water. FeCl_3_-acidified aqueous solutions of ChCl and glycerol were prepared by adding both 20 wt% DI water and 1.32 wt% FeCl_3_·6H_2_O to neat ChCl and glycerol, respectively. Aqueous solution of FeCl_3_ was prepared by dissolving 1.32 wt% FeCl_3_·6H_2_O in DI water.

### 2.3. Pretreatment

Pretreatments of CS were carried out in 120 mL Ace pressure tubes sealed with polytetrafluoroethylene (PTFE) plugs (Ace Glass, Vineland, NJ, USA) at 120 °C in a preheated oil bath for 4 h. The CS concentration was 10 wt% based on the weight of pretreatment solutions, namely, 1.5 g of CS was mixed with 15 g of pretreatment solution. Upon completion of pretreatment, the tube was taken out of the oil bath and cooled to room temperature. Next, 15 mL of ethanol aqueous solution (60% ethanol, *v*/*v*) was added to the pretreatment slurry followed by vacuum-filtration. The retained solid was washed again with 15 mL of ethanol aqueous solution twice to remove residual pretreatment agent. After drying to constant weight at 60 °C, the obtained solid was used for determination of solid recovery, compositional analysis, structure and morphology characterization, and subsequent enzymatic hydrolysis experiments.

The filtrate collected from slurry filtration and washing of pretreated solids was combined and rotary-vacuum evaporated at 40 °C to remove ethanol. The precipitated lignin after ethanol removal was collected by centrifugation at 10,000× *g* for 5 min, washed with 60% (*v*/*v*) ethanol aqueous solution three times and dried under vacuum at 45 °C to constant weight for further use. After removing ethanol, the filtrate was further evaporated at 60 °C to remove excessive water until that its weight was the same as freshly prepared solution used for pretreatment. The recovered pretreatment solutions were directly used for the next round of CS pretreatment.

### 2.4. Enzymatic Hydrolysis

Enzymatic hydrolysis of pretreated solids was conducted in citrate buffer (0.05 M, pH 4.8) with solid concentration at 2 wt%. The loadings of cellulase and β-glucosidase were 30 FPU/g and 60 CBU/g substrate, respectively. Sodium azide (0.02%) were added to the reaction mixture to inhibit microbial contamination. Enzymatic hydrolysis was carried out in a rotary incubator at 50 °C for 48 h at 150 rpm. Samples were taken regularly, filtered through 0.22 μm syringe filter and then analyzed by HPLC.

### 2.5. Analytic Methods

The compositions of CS were determined according to the recommended US National Renewable Energy Laboratory (NREL) protocol [26]. The contents of xylose, glucose and pretreatment by-products (i.e., acetic acid, furfural and 5-hydroxymethylfurfural (HMF)) were measured using Shimadzu LC 20A HPLC (Kyoto, Japan) following the method described by Li, et al. [27]. The FPA and β-glucosidase activity of the used enzyme preparations were determined based on the method of Ghose [28].

The chemical structure, surface morphology and crystalline structure of CS before and after pretreatment were characterized by FT-IR (Nicolet Is50, Thermo Fisher Scientific Co., Waltham, MA, USA), SEM (JSM-6700F, JEOL, Tokyo, Japan) and XRD meter (Smartlab 9, Rigaku Corporation, Tokyo, Japan), respectively. The crystalline index (CrI) was calculated using the following equation according to the method reported by Segal, et al. [29].

CrI = (I_002_ − I_am_)/I_002_ × 100% (1)

I_002_ is the diffraction intensity of the crystallinity peak of cellulose at 2θ ≈ 22.5° and I_am_ is the diffraction intensity of the amorphous region at 2θ ≈ 18°.

### 2.6. Statistical Analysis

Each experiment was performed at least in duplicate with data reported as average values with standard deviations. Statistical analysis was conducted by subjecting the experimental data to one-way analysis of variance (ANOVA) test using Originpro 2018 software (Origin Lab Corporation, Northampton, MA, USA) at 95% confidence level.

## 3. Results and Discussion

### 3.1. Effect of Different Pretreatments on CS Compositions

Five solvents were synthesized and employed to pretreat CS. The compositions of differently pretreated CS are shown in Table 1. Whether without or with addition of water, pretreatment with FeCl_3_ acidified ChCl-glycerol DES preserved the same amount of glucan in the pretreated solids, while acidified aqueous DES resulted in increased solid recovery yield, due to the reduced removal of xylan and lignin compared to acidified neat DES. This indicated that the presence of 20 wt% water deteriorated the performance of acidified DES by decreasing its capability of dissolving xylan and lignin. The role of water in DES pretreatment of cellulosic biomass was reported to be amount-dependent. Kumar, et al. [30] found that addition of 5% water could facilitate lignin removal during rice straw pretreatment with DES consisting of lactic acid and ChCl, attributing to the improved mass transfer due to the reduced viscosity of DES solution with water addition. On the other hand, Chen, et al. [20] observed that aqueous ChCl-glycerol DES containing 20% water led to almost unchanged glucan recovery and decreased removal of xylan and lignin during switchgrass pretreatment compared to neat DES, which was in well agreement with our results. When solitary FeCl_3_ was used for pretreatment, xylan removal was higher, while lignin removal was lower, than acidified DES pretreatment, suggesting that main roles of DES in acidified solution was to removal lignin while preserving xylan. FeCl_3_ acidified aqueous glycerol (80 wt%) pretreatment led to the same removals of glucan and xylan but higher delignification than FeCl_3_ pretreatment, implying the facilitating effect of glycerol on removing lignin selectively. Pretreatment with combined FeCl_3_ and ChCl yielded the best results in terms of preserving the highest level of glucan while removing the highest amount of xylan and lignin among the five tested solvents, resulting in pretreated solids enriched with cellulose. Those meant that ChCl played multiple positive roles in FeCl_3_-facilited pretreatment of cellulosic materials compared to DES and glycerol.

In summary, our results showed that acidified aqueous ChCl was more effective in deconstruction of lignocellulose than acidified aqueous glycerol and neat/aqueous DES composed of ChCl and glycerol. This showed that ChCl, as a cheap animal feed ingredient, is a potential agent used for biomass pretreatment.

### 3.2. Enzymatic Hydrolysis of Differently Pretreated Solids

**Figure 1:**
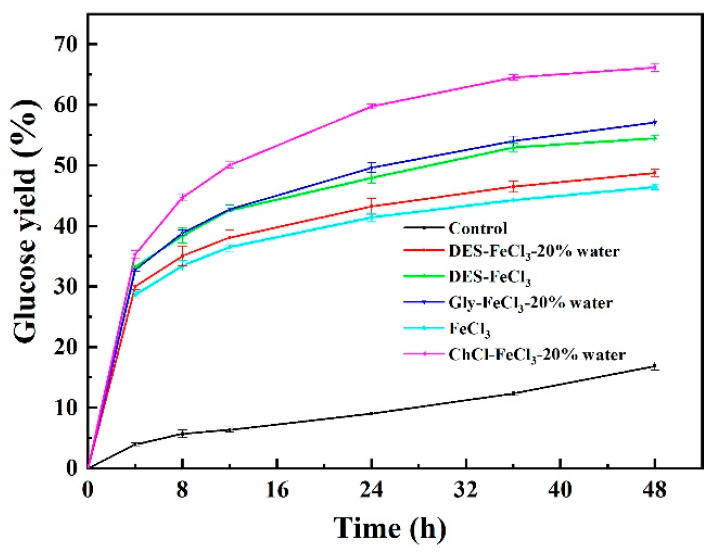
Enzymatic digestibility of untreated and the pretreated biomass with different pretreatment solvents.

Five different pretreatments produced solids with different compositions of cellulose, xylan and lignin. Enzymatic digestibility of pretreated solids was performed to evaluate and compare the pretreatment efficiency of five solvents. Figure 1 shows the glucose yield from enzymatic hydrolysis of differently pretreated solids. It was obvious that raw CS had the lowest digestibility over the time course of enzymatic saccharification due to its high recalcitrance. Enzymatic digestibility of CS was greatly improved by pretreatment and the enhancement degree was dependent on the applied pretreatment solution. FeCl_3_-assisted ChCl pretreatment demonstrated the highest glucose yield of 66.18% after 48 h enzymatic hydrolysis, followed by pretreatment with acidified aqueous glycerol (57.10%) and acidified neat DES (54.47%). Pretreatment with FeCl_3_-facilited aqueous DES and single FeCl_3_ yielded to low levels of enzymatic cellulose hydrolysis with 48 h glucose yield being 48.78% and 46.43%, respectively.

**Figure 2:**
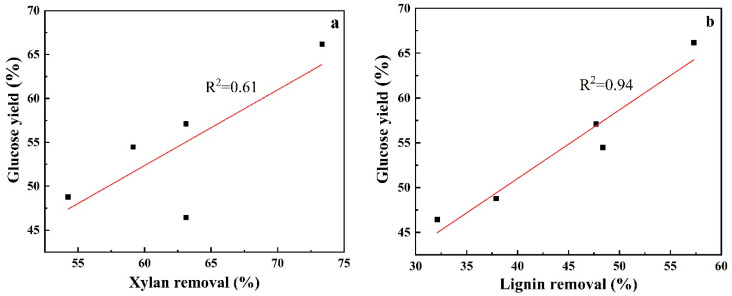
Correlation between enzyme digestibility of pretreated solids and removals of xylan (**a**) and lignin (**b**).

It can be seen from above enzymatic digestibility dada that different pretreatments resulted in differently improved enzymatic conversion of pretreated solids and the highest glucose yield was obtained from enzymatic hydrolysis of CS pretreated with acidified aqueous ChCl solution. It was necessary to explore the main factors responsible for this phenomenon. As discussed in Section 3.1, different removals of xylan and lignin were observed in the tested five pretreatments, therefore, there is a reason to believe that enzymatic hydrolysis efficiency of pretreated CS was likely dependent on the removals of xylan and lignin. To confirm this assumption, the removals of xylan and lignin after pretreatments were plotted against glucose yields from enzymatic conversion of correspondingly pretreated CS at 48 h. As presented in Figure 2, xylan removal showed a weak correlation to glucose yield as indicated by a low R^2^ value of 0.61. In sharp contrast to xylan removal, lignin removal demonstrated a highly linear relationship (R^2^ = 0.94) with corresponding enzymatic digestibility, suggesting that delignification affect enzymatic cellulose conversion more significantly than xylan removal. The fact that depolymerization of xylan and lignin after biomass pretreatment favored subsequent enzymatic hydrolysis might be due to the more contact of cellulase with cellulose fraction of pretreated solids because xylan and lignin surrounds cellulosic fiber and acts a physical barrier preventing the access of cellulase to cellulose [31,32]. While the more important effect of delignification on enzymatic hydrolysis than that of xylan removal might be caused by the more roles that lignin plays in enzymatic cellulose conversion. On the one hand, similar to xylan, lignin could serve as a barrier to cellulase. On the other hand, lignin contained in pretreated solids could adsorb cellulase non-productively during enzymatic depolymerization of pretreated solids, reducing the amount of cellulase available for hydrolyzing cellulosic substrate [33,34]. Additionally, it was reported that during high-temperature acid pretreatments, the released lignin could mitigate and re-deposit on the exposed cellulose surface, limiting the attack of cellulase towards cellulose [33,35].

### 3.3. Characterization of Differently Pretreated Solid Residues

In order to explore how pretreatment changed the physico-chemical structure of CS and improved subsequent enzymatic saccharification, the surface morphology and chemical structure of pretreated solids was characterized by FT-IR, SEM and XRD so as to better understand the acting mechanisms of pretreatment.

**Figure 3:**
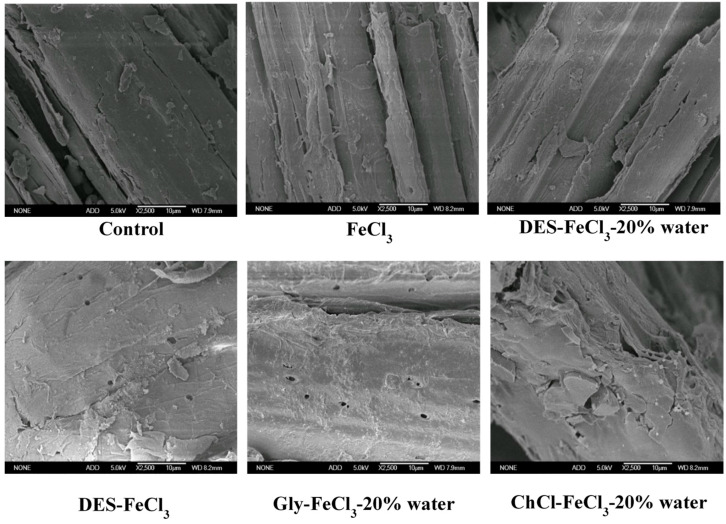
Scanning electron microscopy (SEM) images of CS before and after pretreatments.

Figure 3 shows the SEM images of CS before and after pretreatments. The surface of untreated CS is relatively rigid and compact, while the surface of pretreated samples became rough and porous. Noticeably, the orderly structure of raw biomass is destroyed after pretreatments. It was presumed that the deconstruction of surface structure of pretreated CS was most likely due to the fact that the pretreatment agents attacked and depolymerized the fractions of lignin and hemicellulose. By comparison, acidified aqueous ChCl pretreatment led to the most fragmented structure of pretreated solids among the tested pretreatments, again corroborating its best pretreatment performance. Structure fragmentation and removal of non-cellulosic components exposed cellulose surface and increased the accessibility of enzymes, thereby significantly increasing the rate and yield of enzymatic conversion of cellulose to glucose.

**Figure 4:**
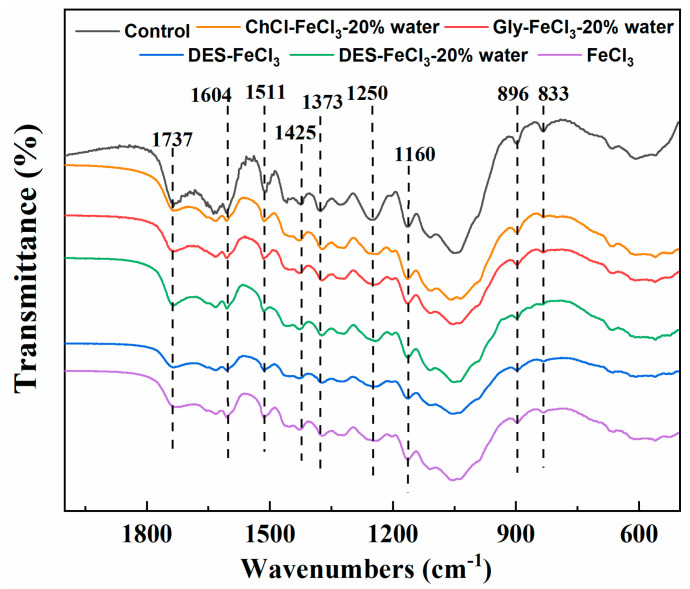
Fourier transform infrared (FTIR) spectra of CS before and after pretreatments.

Figure 4 displays the FT-IR spectra of CS before and after pretreatment. The typical infrared bands were assigned according to the previous literatures [36,37,38,39] unless otherwise specified. The intensities of lignin-associated adsorption peaks appeared at 1604 cm^−1^ and 1511 cm^−1^ (both corresponding to aromatic benzene ring skeleton stretching), 1250 cm^−1^ (C–O stretching of the lignin) and 833 cm^−1^ (plane vibration in syringyl lignin). It was obvious that those peak intensities became weaker after pretreatment, implying partial removal of lignin. The bands at 1373 and 1425 cm^−1^ are related to C–H deformation and CH_2_ scissor vibration in cellulose and hemicellulose, respectively. The bands at 1160 and 896 cm^−1^ band corresponds to C–O–C stretching at the β-(1→4)-glycosidic linkages in cellulose and hemicellulose [40]. The decrease in the intensity of hemicellulose characteristic band at 1736 cm^−1^ after pretreatments confirmed the removal of hemicellulose. By observing closely, it could be found that the weakest intensities of characteristic absorption bands for lignin and hemicellulose were from pretreatment with acidified aqueous ChCl solution, which meant that acidified aqueous ChCl pretreatment removed the highest amount of lignin and hemicellulose among the five pretreatments. These results were in well agreement with the data shown in Table 1.

**Figure 5:**
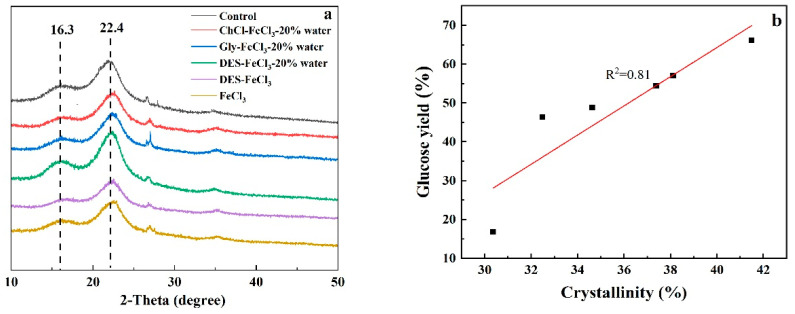
(**a**) X-ray diffraction (XRD) patterns of CS before and after pretreatments; (**b**) correlation between CrI and enzymatic cellulose hydrolysis.

Figure 5a presents the XRD patterns of raw and five pretreated solid residues and Table 2 showed the calculated CrI from the obtained XRD data. The CrI values of the raw material was determined to be 30.36%, while CrI values increased to about 34% for pretreatments with both FeCl_3_ and acidified aqueous DES, and to approximately 38% for pretreatments with acidified solutions of both glycerol and neat DES. The highest CrI value of 41.49% was attained from acidified aqueous ChCl pretreated samples. CrI is a variable that measures the relative percentage of crystalline cellulose in the total solid. The observed increase in the CrI values of pretreated solids could be explained by the large removal of amorphous hemicellulose and lignin as well as destruction of amorphous cellulose. By plotting CrI values of differently pretreated CS against corresponding enzymatic digestibility, it could be found that there was a good correlation between them (Figure 5b), implying that CrI values of pretreated solid was an effective indicator for predicting its enzymatic conversion in the present study.

### 3.4. Monosaccharides and Polysaccharide-derived Inhibitors in Pretreatment Liquids

As reviewed by Kim [41] and Kumar, et al. [42], during thermochemical acid pretreatment of cellulosic biomass, the cellulose and hemicellulose fractions were depolymerized to oligomeric and monomeric saccharides, which could be further dehydrated to generate furan derivatives, such as furfural and 5-hydroxymethyl furfural (HMF). Dehydration of acetyl group of hemicellulose resulted in formation of acetic acid. Depolymerization of lignin also leads to generation of fractured lignin with low molecular-weights and various phenolic compounds. These pretreatment by-products are reported to be potent inhibitors for (hemi)cellulolytic enzymes and fermentative microorganisms.

**Figure 6:**
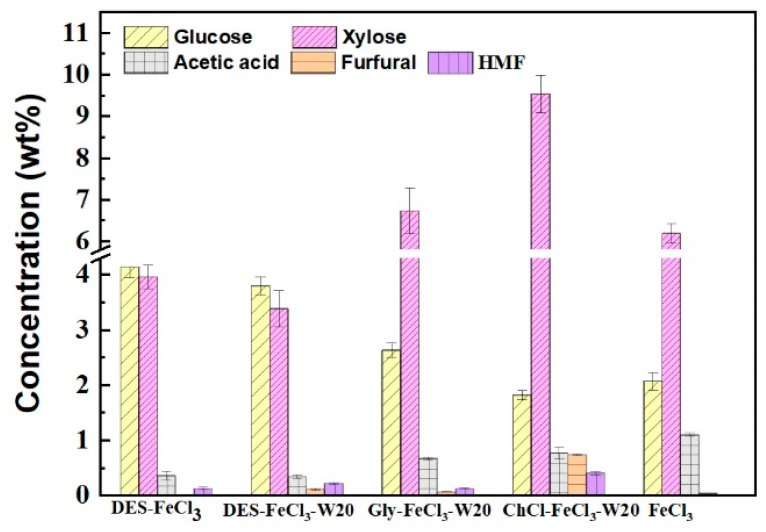
The contents of monosaccharide and polysaccharide-derived inhibitors present in different pretreatment streams. W20 stands for addition of 20 wt% water.

The contents of monosaccharide and polysaccharide-derived inhibitors present in different pretreatment streams are analyzed. As shown in Figure 6, xylose and glucose were found to be the main monosaccharides in all the pretreatment solutions, but the concentration ratio of xylose to glucose were different for different pretreatments. Nearly equivalent amounts of xylose and glucose were detected in the pretreatment solution of acidified neat and aqueous DES, whereas higher amounts of xylose than glucose were attained in pretreatment liquids of acidified aqueous ChCl and glycerol, as well as solitary FeCl_3_. Acetic acid was the highest concentration of inhibitor in all the pretreatment liquids. Its concentration was in the following order: single FeCl_3_ > ChCl-FeCl_3_ > glycerol-FeCl_3_ > neat DES-FeCl_3_ > aqueous DES-FeCl_3_. Furfural and HMF with varied concentrations were also present in the five tested pretreatment solutions. HMF was the second highest concentration of inhibitor in the pretreatment liquors of acidified neat and aqueous DES, as well as acidified aqueous glycerol, while for acidified aqueous ChCl pretreatment, the contents of both HMF and furfural were the highest among the obtained five pretreatment solutions. In contrary to the acidified DES and glycerol pretreatment, the content of furfural was higher than HMF for pretreatments with acidified aqueous ChCl. Solitary FeCl_3_ pretreatments gave relatively lower levels of furfural and HMF than other pretreatments. It was worthwhile noting that the contents of those compounds were significantly lower than other commonly used chemical pretreatments, such as acid, steam explosion and wet oxidation [43,44].

### 3.5. Recyclability and Reusability of Acidified Solutions

**Figure 7:**
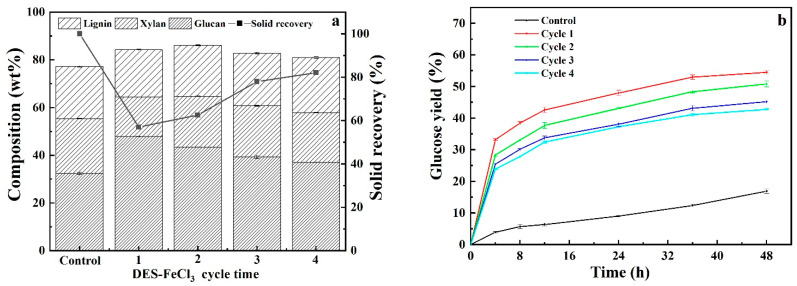
Compositional analysis (**a**) and enzymatic digestibility (**b**) of CS pretreated by recycled acidified neat ChCl-glycerol DES.

**Figure 8:**
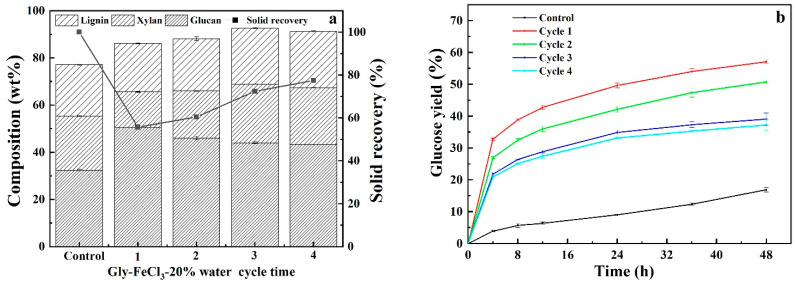
Compositional analysis (**a**) and enzymatic digestibility (**b**) of CS pretreated by recycled acidified glycerol solution.

**Figure 9:**
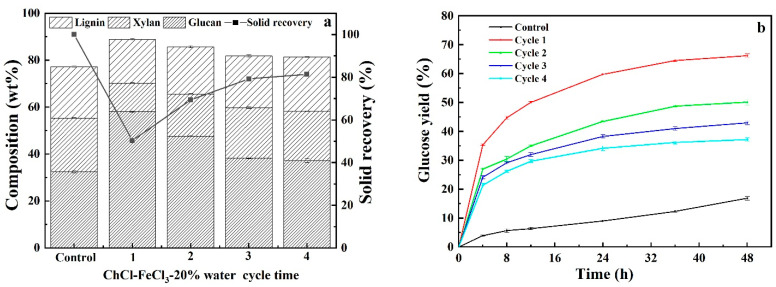
Compositional analysis (**a**) and enzymatic digestibility (**b**) of CS pretreated by recycled acidified ChCl solution.

**Figure 10:**
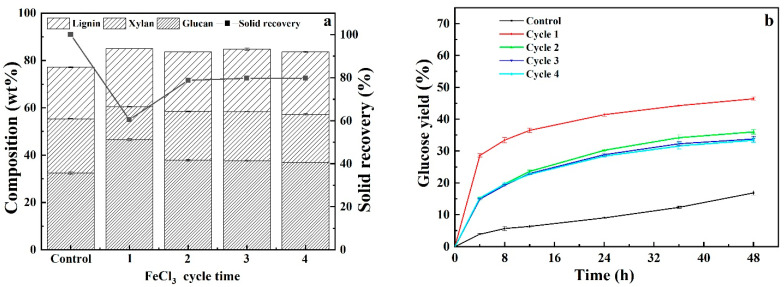
Compositional analysis (**a**) and enzymatic digestibility (**b**) of CS pretreated by recycled FeCl_3_ solution.

Repeated use of pretreatment solution for multiple rounds of CS pretreatment can substantially save pretreatment cost. In light of this, the recoverability and reusability of acidified solutions of neat DES, glycerol and ChCl and single FeCl_3_ solution were evaluated by regenerating pretreatment solutions as follows: After each pretreatment, the pretreatment slurry was filtrated, and the solid residues was washed with aqueous ethanol solution three times. The filtrate and washing solution were combined and vacuum-evaporated to remove ethanol to obtained precipitated lignin. Thereafter, evaporation was continually carried out to remove water until the weight of pretreatment liquor was the same as that of freshly prepared counterpart. The recycled pretreatment liquor was used for subsequent round of CS pretreatment and recyclability experiments were performed for four cycles. It should be noted that the recycled pretreatment liquors contained impurities because, as shown in Figure 6, carbohydrate and pretreatment by-products that cannot be removed via evaporation could be accumulated with increasing cycles. As can be seen from Figure 7, Figure 8, Figure 9 and Figure 10, it was observed that removals of glucan were dramatically different from that of xylan and lignin for four pretreatments. Comparable removals of glucan were observed in the first two recycles and then significantly decreased in the third and fourth runs, where both also demonstrated comparable glucan removals, for pretreatments with DES-FeCl_3_, glycerol-FeCl_3_ and ChCl-FeCl_3_. However, for individual FeCl_3_ pretreatment, glucan removal significantly reduced after first run and then varied with a small fluctuation. After four rounds of pretreatment, the glucan removals decreased by 62.03%, 100%, 32.96% and 30.68% for FeCl_3_ facilitated pretreatments with neat DES, glycerol and ChCl and individual FeCl_3_ pretreatment, respectively. When it came to removals of xylan and lignin, more than 50% reductions in removals of xylan and lignin were observed after two pretreatment cycles and thereafter removals of lignin and xylose remained slightly changed for the tested four pretreatments. After four times of repeated use of pretreatment agents, the removals of xylan deceased by 56.36%, 70.75%, 65.19% and 53.06% for pretreatments with DES-FeCl_3_, glycerol-FeCl_3_, ChCl-FeCl_3_ and single FeCl_3_ solutions, respectively, whereas the reductions in lignin removals were calculated to be 71.23%, 53.42%, 75.11% and 39.39%, for above mentioned four pretreatments, respectively. In tandem with decreases in the removals of lignin and xylan, the glucose yields after 48 h enzymatic hydrolysis decreased by 22.07%, 34.80%, 43.80% and 28.04% for pretreatments with acidified solutions of neat DES, aqueous glycerol, aqueous ChCl and single FeCl_3_, respectively.

The performance of recycled pretreatment solutions deteriorated with increasing recycle times. The plausible reason for this phenomenon was that acidity of the pretreatment solution gradually decreased as the measured pH of the pretreatment solution increased from 2.19 to 4.4 for DES-FeCl_3_ pretreatment, from 1.05 to 3.84 for Glycerol-FeCl_3_ pretreatment, from 1.77 to 4.59 for ChCl-FeCl_3_ pretreatment and from 1.75 to 3.67 for single FeCl_3_ pretreatment. The increase in the pH of all the tested pretreatment solution was likely due the loss of lewis acid Fe^3+^ caused by formation of complexes with hemicellulose and lignin [45]. Similar result was also reported by Chen, et al. [25], who evaluated the recyclability of H_2_SO_4_-acidified aqueous ChCl during pretreatment of switchgrass. They observed significantly decreased pretreatment effectiveness when the solution was recycled twice. After addition with acid to lower the pH of the solution to that of freshly prepared one in the third run, the pretreatment performance of recycled solution was greatly recovered to the level of the first two cycles. However, even with adjusting pH, the effectiveness of the fifth cycle of pretreatment still decreased compared to previous runs. They attributed the reason to the accumulated impurities such as pretreatment by-products in the recycled liquid, as well as minor solvent loss. Wang, et al. [21] evaluated the reusability of FeCl_3_ assisted DES in pretreatment of Hybrid Pennisetum and found that cellulose content in the pretreated solid decreased from 80.94% of the first cycle to 73.76% of the fourth cycle. They deduced that reduction in both the acidity and hydrogen-bond interaction in solution as well as impurities in DES after each cycle could explain the decreased cellulose content with increasing pretreatment recycles. Unexpectedly, the removals of xylan and lignin did no change significantly after four cycles, which was completely contrary to the experimental results in the present study. This could be explained by the difference in the applied pretreatment conditions and/or cellulosic substrate.

## 4. Conclusions

In this work, corn stover (CS) pretreatments with FeCl_3_-acidified solution of aqueous choline chloride (ChCl), aqueous glycerol and ChCl-glycerol deep eutectic solvent (DES) were examined and compared with single FeCl_3_ pretreatment as control. It was found that acidified aqueous ChCl pretreatment removed the highest amount of lignin (57.29%) and xylan (73.35%), followed by acidified aqueous glycerol pretreatment, which removed 47.70% lignin and 63.11% xylan. Acidified neat DES pretreatment was less effective with delignification and xylan removal being 48.35% and 59.15%, respectively. Addition of 20 wt% water to acidified neat DES further decreased the removals of lignin and xylan. Correspondingly, enzymatic cellulose saccharification followed the order of aqueous ChCl > aqueous glycerol > neat DES > aqueous DES. Correlation analysis showed that digestibility of pretreated solids was strongly correlated to delignification and CrI values but poorly correlated to xylan removal. Surface morphology and chemical structure of differently pretreated CS were characterized and compared with that of raw CS, results showed that variations in intensities of Fourier transform infrared (FTIR) characteristic peaks and increase in crystallinity index calculated from X-ray diffraction (XRD) patterns confirmed the enrichment of cellulose and removal of lignin and xylan. Analysis of pretreatment solutions demonstrated that acidified aqueous ChCl pretreatment yielded the higher contents of acetic acid and furan inhibitors than acidified DES and glycerol pretreatments. Moreover, after repeated use of pretreatment solution for 4 times, the least decease in cellulose saccharification was observed from acidified neat DES pretreatment (22.07%) and the highest decrease from acidified aqueous ChCl pretreatment (43.80%). These findings indicated that ChCl and glycerol solutions were more effective for lignocellulose pretreatment than ChCl-glycerol DES, albeit with poor reusability.

## Figures and Tables

**Table 1:** Effect of different pretreatment solvents on chemical composition of corn stover (CS).

Pretreatment Solvent	Solid Recovery (%)	Removal (%)		
		Glucan	Xylan	Lignin
ChCl-Gly-FeCl_3_	56.98 ± 0.86	15.75 ± 0.62	59.15 ± 0.28	48.35 ± 0.75
ChCl-Gly-FeCl_3_-20% water	60.66 ± 1.53	15.41 ± 1.48	54.25 ± 1.38	37.90 ± 0.75
FeCl_3_	60.51 ± 0.98	13.18 ± 0.83	63.12 ± 0.26	32.12 ± 0.18
Gly-FeCl_3_-20% water	55.71 ± 1.12	13.23 ± 0.41	63.11 ± 0.71	47.70 ± 0.69
ChCl-FeCl_3_-20% water	50.31 ± 1.34	9.83 ± 0.48	73.35 ± 0.82	57.29 ± 0.83

**Table 2:** Crystallinity of pretreated and untreated samples.

Pretreatment Solvent	Crystallinity (%)	48 h Glucose Yield (%)
Control	30.36	16.87 ± 0.68
FeCl_3_	32.49	46.43 ± 0.47
DES-FeCl_3_-20% water	34.63	48.78 ± 0.63
DES-FeCl_3_	37.38	54.47 ± 0.52
Gly-FeCl_3_-20% water	38.11	57.10 ± 0.60
ChCl-FeCl_3_-20% water	41.49	66.18 ± 0.66

## Data Availability

The data presented in this study are available on request from the corresponding author.

## References

[1] Arevalo-Gallegos A., Ahmad Z., Asgher M., Parra-Saldivar R., Iqbal H.M.N. (2017). Lignocellulose: A sustainable material to produce value-added products with a zero waste approach—A review. Int. J. Biol. Macromol..

[2] Jahirul M.I., Rasul M.G., Chowdhury A.A., Ashwath N. (2012). Biofuels Production through Biomass Pyrolysis —A Technological Review. Energ..

[3] Iqbal H.M.N., Kyazze G., Keshavarz T. (2013). Advances in the valorization of lignocellulosic materials by biotechnology: An overview. BioResources.

[4] Nguyen T.Y., Cai C.M., Kumar R., Wyman C.E. (2017). Overcoming factors limiting high-solids fermentation of lignocellulosic biomass to ethanol. Proc. Natl. Acad. Sci. USA.

[5] Taherzadeh M.J., Karimi K. (2008). Pretreatment of Lignocellulosic Wastes to Improve Ethanol and Biogas Production: A Review. Int. J. Mol. Sci..

[6] Kucharska K., Rybarczyk P., Hołowacz I., Łukajtis R., Glinka M., Kamiński M. (2018). Pretreatment of Lignocellulosic Materials as Substrates for Fermentation Processes. Molecules.

[7] Rastogi M., Shrivastava S. (2017). Recent advances in second generation bioethanol production: An insight to pretreatment, saccharification and fermentation processes. Renew. Sust. Energ. Rev..

[8] Kalhor P., Ghandi K. (2019). Deep Eutectic Solvents for Pretreatment, Extraction, and Catalysis of Biomass and Food Waste. Molecules.

[9] Chen Y., Mu T. (2019). Application of deep eutectic solvents in biomass pretreatment and conversion. Green Energy Environ..

[10] Zhang Q., Vigier K.D.O., Royer S., Jerome F. (2012). Deep eutectic solvents: Syntheses, properties and applications. Chem. Soc. Rev..

[11] Smith E.L., Abbott A.P., Ryder K.S. (2014). Deep eutectic solvents (DESs) and their applications. Chem. Rev..

[12] Grillo G., Calcio Gaudino E., Rosa R., Leonelli C., Timonina A., Grygiškis S., Tabasso S., Cravotto G. (2021). Green Deep Eutectic Solvents for Microwave-Assisted Biomass Delignification and Valorisation. Molecules.

[13] Li C., Huang C., Zhao Y., Zheng C., Su H., Zhang L., Luo W., Zhao H., Wang S., Huang L.-J. (2021). Effect of Choline-Based Deep Eutectic Solvent Pretreatment on the Structure of Cellulose and Lignin in Bagasse. Processes.

[14] Satlewal A., Agrawal R., Bhagia S., Sangoro J., Ragauskas A.J. (2018). Natural deep eutectic solvents for lignocellulosic biomass pretreatment: Recent developments, challenges and novel opportunities. Biotechnol. Adv..

[15] Xu H., Peng J., Kong Y., Liu Y., Su Z., Li B., Song X., Liu S., Tian W. (2020). Key process parameters for deep eutectic solvents pretreatment of lignocellulosic biomass materials: A review. Bioresour. Technol..

[16] Procentese A., Raganati F., Olivieri G., Russo M.E., Rehmann L., Marzocchella A. (2017). Low-energy biomass pretreatment with deep eutectic solvents for bio-butanol production. Bioresour. Technol..

[17] Fang C., Thomsen M.H., Frankær C.G., Brudecki G.P., Schmidt J.E., AlNashef I.M. (2017). Reviving pretreatment effectiveness of deep eutectic solvents on lignocellulosic date palm residues by prior recalcitrance reduction. Ind. Eng. Chem. Res..

[18] Procentese A., Johnson E., Orr V., Campanile A.G., Wood J.A., Marzocchella A., Rehmann L. (2015). Deep eutectic solvent pretreatment and subsequent saccharification of corncob. Bioresour. Technol..

[19] Alvarez-Vasco C., Ma R., Quintero M., Guo M., Geleynse S., Ramasamy K.K., Wolcott M., Zhang X. (2016). Unique low-molecular-weight lignin with high purity extracted from wood by deep eutectic solvents (DES): A source of lignin for valorization. Green Chem..

[20] Chen Z., Reznicek W.D., Wan C.X. (2018). Deep eutectic solvent pretreatment enabling full utilization of switchgrass. Bioresour. Technol..

[21] Wang Z.K., Li H., Lin X.C., Tang L., Chen J.J., Mo J.W., Yu R.S., Shen X.J. (2020). Novel recyclable deep eutectic solvent boost biomass pretreatment for enzymatic hydrolysis. Bioresour. Technol..

[22] Ferreira J.A., Taherzadeh M.J. (2020). Improving the economy of lignocellulose-based biorefineries with organosolv pretreatment. Bioresour. Technol..

[23] Tang S., Dong Q., Fang Z., Cong W.-j., Miao Z.-d. (2019). High-concentrated substrate enzymatic hydrolysis of pretreated rice straw with glycerol and aluminum chloride at low cellulase loadings. Bioresour. Technol..

[24] Santana J.C., Souza Abud A.K., Wisniewski A., Navickiene S., Romão L.P.C. (2020). Optimization of an organosolv method using glycerol with iron catalysts for the pretreatment of water hyacinth. Biomass Bioenerg..

[25] Chen Z., Reznicek W.D., Wan C. (2018). Aqueous Choline Chloride: A Novel Solvent for Switchgrass Fractionation and Subsequent Hemicellulose Conversion into Furfural. ACS Sustain. Chem. Eng..

[26] Sluiter A., Hames B., Scarlata C., Sluiter J., Templeton D., Crocker D. (2008). Determination of structural carbohydrates and lignin in biomass—NREL/TP-510-42618. Lab. Anal. Proced. (LAP).

[27] Li Y., Qi B., Wan Y. (2020). Separation of monosaccharides from pretreatment inhibitors by nanofiltration in lignocellulosic hydrolysate: Fouling mitigation by activated carbon adsorption. Biomass Bioenerg..

[28] Ghose T. (1987). Measurement of cellulase activities. Pure Appl. Chem..

[29] Segal L., Creely J., Martin Jr A., Conrad C. (1959). An empirical method for estimating the degree of crystallinity of native cellulose using the X-ray diffractometer. Text. Res. J..

[30] Kumar A.K., Parikh B.S., Pravakar M. (2016). Natural deep eutectic solvent mediated pretreatment of rice straw: Bioanalytical characterization of lignin extract and enzymatic hydrolysis of pretreated biomass residue. Environ. Sci. Pollut. Res..

[31] Tarasov D., Leitch M., Fatehi P. (2018). Lignin–carbohydrate complexes: Properties, applications, analyses, and methods of extraction: A review. Biotechnol. Biofuels.

[32] Cai J., He Y., Yu X., Banks S.W., Yang Y., Zhang X., Yu Y., Liu R., Bridgwater A.V. (2017). Review of physicochemical properties and analytical characterization of lignocellulosic biomass. Renew. Sust. Energ. Rev..

[33] Yoo C.G., Meng X., Pu Y., Ragauskas A.J. (2020). The critical role of lignin in lignocellulosic biomass conversion and recent pretreatment strategies: A comprehensive review. Bioresour. Technol..

[34] Saini J.K., Patel A.K., Adsul M., Singhania R.R. (2016). Cellulase adsorption on lignin: A roadblock for economic hydrolysis of biomass. Renew. Energ..

[35] Zakaria M.R., Hirata S., Hassan M.A. (2015). Hydrothermal pretreatment enhanced enzymatic hydrolysis and glucose production from oil palm biomass. Bioresour. Technol..

[36] El Hage R., Brosse N., Chrusciel L., Sanchez C., Sannigrahi P., Ragauskas A. (2009). Characterization of milled wood lignin and ethanol organosolv lignin from miscanthus. Polym. Degrad. Stab..

[37] Cai Q., Fan Z.S., Chen J.B., Guo W.J., Ma F., Sun S.Q., Hu L.M., Zhou Q. (2018). Dissolving process of bamboo powder analyzed by FT-IR spectroscopy. J. Mol. Struct..

[38] Zhang C.W., Xia S.Q., Ma P.S. (2016). Facile pretreatment of lignocellulosic biomass using deep eutectic solvents. Bioresour. Technol..

[39] Li X., Wei Y., Xu J., Xu N., He Y. (2018). Quantitative visualization of lignocellulose components in transverse sections of moso bamboo based on FTIR macro- and micro-spectroscopy coupled with chemometrics. Biotechnol. Biofuels.

[40] Zhuang J., Li M., Pu Y., Ragauskas A.J., Yoo C.G. (2020). Observation of Potential Contaminants in Processed Biomass Using Fourier Transform Infrared Spectroscopy. Appl. Sci..

[41] Kim D. (2018). Physico-Chemical Conversion of Lignocellulose: Inhibitor Effects and Detoxification Strategies: A Mini Review. Molecules.

[42] Kumar V., Yadav S.K., Kumar J., Ahluwalia V. (2020). A critical review on current strategies and trends employed for removal of inhibitors and toxic materials generated during biomass pretreatment. Bioresour. Technol..

[43] van der Pol E.C., Bakker R.R., Baets P., Eggink G. (2014). By-products resulting from lignocellulose pretreatment and their inhibitory effect on fermentations for (bio)chemicals and fuels. Appl. Microbiol. Biotechnol..

[44] Qin L., Liu L., Li W.-C., Zhu J.-Q., Li B.-Z., Yuan Y.-J. (2016). Evaluation of soluble fraction and enzymatic residual fraction of dilute dry acid, ethylenediamine, and steam explosion pretreated corn stover on the enzymatic hydrolysis of cellulose. Bioresour. Technol..

[45] Román-Leshkov Y., Davis M.E. (2011). Activation of Carbonyl-Containing Molecules with Solid Lewis Acids in Aqueous Media. ACS Catalysis.

